# Reanalysis of Exome Data Identifies Novel *SLC25A46* Variants Associated with Leigh Syndrome

**DOI:** 10.3390/jpm11121277

**Published:** 2021-12-02

**Authors:** Qifei Li, Jill A. Madden, Jasmine Lin, Jiahai Shi, Samantha M. Rosen, Klaus Schmitz-Abe, Pankaj B. Agrawal

**Affiliations:** 1 Division of Newborn Medicine, Boston Children’s Hospital, Harvard Medical School, Boston, MA 02115, USA; Qifei.Li@childrens.harvard.edu (Q.L.); Jasmine.Lin@childrens.harvard.edu (J.L.); Samantha.Rosen@childrens.harvard.edu (S.M.R.); Klaus.Schmitz-Abe@childrens.harvard.edu (K.S.-A.); 2 The Manton Center for Orphan Disease Research, Boston Children’s Hospital, Harvard Medical School, Boston, MA 02115, USA; Jill.Madden@childrens.harvard.edu; 3 Division of Genetics and Genomics, Boston Children’s Hospital, Harvard Medical School, Boston, MA 02115, USA; 4 Department of Biomedical Sciences, City University of Hong Kong, Kowloon, Hong Kong, China; jiahai.shi@cityu.edu.hk

**Keywords:** *SLC25A46*, mitochondria, optic atrophy, Leigh syndrome, reanalysis

## Abstract

*SLC25A46* (solute carrier family 25 member 46) mutations have been linked to various neurological diseases with recessive inheritance, including Leigh syndrome, optic atrophy, and lethal congenital pontocerebellar hypoplasia. SLC25A46 is expressed in the outer membrane of mitochondria, where it plays a critical role in mitochondrial dynamics. A deceased 7-month-old female infant was suspected to have Leigh syndrome. Clinical exome sequencing was non-diagnostic, but research reanalysis of the sequencing data identified two novel variants in *SLC25A46*: a missense (c.1039C>T, p.Arg347Cys; NM_138773, hg19) and a donor splice region variant (c.283+5G>A) in intron 1. Both variants were predicted to be damaging. Sanger sequencing of cDNA detected a single missense allele in the patient compared to control, and the *SLC25A46* transcript levels were also reduced due to the splice region variant. Additionally, Western blot analysis of whole-cell lysate showed a decrease of SLC25A46 expression in proband fibroblasts, relative to control cells. Further, analysis of mitochondrial morphology revealed evidence of increased fragmentation of the mitochondrial network in proband fibroblasts, compared to control cells. Collectively, our findings suggest that these novel variants in *SLC24A46*, the donor splice one and the missense variant, are the cause of the neurological phenotype in this proband.

## 1. Introduction

The solute carrier family 25 member 46 (SLC25A46), is an integral mitochondrial outer membrane protein and a member of the mitochondrial solute carrier family SLC25 [1]. Fifty three different SLC25 proteins have been identified in humans and a majority are responsible for the transport of diverse metabolites across the inner mitochondrial membrane as a vital step to an array of metabolic pathways [2,3]. In contrast to other members, SLC25A46 localizes to the outer mitochondrial membrane and has not demonstrated a transport activity [1,4]. Instead, SLC25A46 interacts with mitofilin, optic atrophy 1 (OPA1), mitofusin 2 (MFN2), and the mitochondrial cristae organizing complex (MICOS) proteins to maintain mitochondrial cristae structure and dynamics [1,5]. SLC25A46 has also been suggested to play a key role in lipid homeostasis by interacting with the endoplasmic reticulum membrane protein complex (EMC) to help facilitate the transfer of phospholipids from the endoplasmic reticulum (ER) to the mitochondria [5,6].

Loss of SLC25A46 can result in mitochondrial hyperfusion, altered mitochondrial architecture and ER morphology, and changes to the phospholipid composition of the mitochondrial membranes [1]. This can ultimately lead to altered mitochondrial dynamics and metabolic dysregulation [7]. Variants in *SLC25A46* were first reported as disease causing in 2015 [1], and since then, several mutations in this gene have been pathologically linked to a range of neurological diseases such as optic atrophy, Leigh syndrome, axonal Charcot–Marie–Tooth disease, and lethal congenital pontocerebellar hypoplasia [1,5,8].

Here, we report the identification of two novel *SLC25A46* variants (NM_138773.2, hg19) following reanalysis of trio exome (ES) data from a deceased seven-month-old female proband with suspected Leigh syndrome: a missense variant (c.1039C>T, p.Arg347Cys) inherited from the mother and a variant in the donor splice region of intron 1 (c.283+5G>A) inherited from the father. We performed transcript and protein analysis on proband cells, used an in silico approach, and linked the proband’s neurological phenotype to the two identified variants in *SLC25A46*.

## 2. Materials and Methods

Clinical evaluation: Informed consent was obtained in accordance with the ethical standards of the participating Institutional Review Board at Boston Children’s Hospital. Clinical records of patients were then reviewed.

Cell Culture: Proband and control fibroblast cells were obtained from the clinical laboratory. Cells were grown in high-glucose DMEM (cat# 11995-073, ThermoFisher Scientific, Waltham, MA, USA) supplemented with 15% fetal bovine serum (cat. number S11550, Atlanta Biological, Minneapolis, MN, USA) and 1% Penicillin-Streptomycin (cat. number 100378-016, ThermoFisher Scientific, Waltham, MA, USA) at 37 °C in an atmosphere of 5% CO_2_. The cell medium was changed every two days. Cells were passaged upon confluency according to standard protocol.

Reverse transcriptase (RT) PCR analysis: Total RNA was isolated from proband and control fibroblasts using the Qiagen RNeasy kit (cat. number 74104, Qiagen, Germantown, MD, USA) according to the manufacturer’s protocol. One µg of total RNA was used to generate complementary DNA (cDNA) using the SuperScript™ IV First-Strand Synthesis System and random hexamers (cat. number 18091050, ThermoFisher Scientific, Waltham, MA, USA). RT reaction was performed at a final volume of 25 µL using DreamTaq polymerase (cat. number EP0701, ThermoFisher Scientific, Waltham, MA, USA) and standard PCR conditions for all amplifications: initial denaturation of 2 min at 94 °C; 30 cycles of 30 s at 94 °C, 30 s at 52 °C, 1 min at 72 °C, and final extension of 5 min at 72 °C. For real-time quantitative PCR (qRT-PCR) primers: (1) Exon1-Exon3 F: GGAGGAACCCTTTTCCAGTG, R: AATGCAAGGATGTGCCAATA; (2) Exon8 F: CCCTGTGCAGAGTATGTTGG, R: AATGTGAAGGCGGTGCAAAA. *TBP* and *RNF111* as the reference genes for normalization. *TBP* F: CACGAACCACGGCACTGATT, R: TTTTCTTGCTGCCAGTCTGGAC; *RNF111* F: GCAGAATGCAGCAGAAGTTG, R: CCATTCTTGCAGAAGTGGTTG.

Western blot analysis: Fibroblasts from proband and two control cells were grown to 3 × 10^6^ cells. Protein lysates were prepared by lysing cells in RIPA buffer (cat. number 89901, ThermoFisher Scientific, Waltham, MA, USA) with protease and phosphatase inhibitor (cat. number 78440, ThermoFisher Scientific, Waltham, MA, USA) and 5 mM EDTA for 30 min on ice, followed by centrifugation at maximum speed and 4 °C for 20 min. Protein measurements were performed using the Pierce BCA assay kit (cat# 23225, ThermoFisher Scientific, Waltham, MA, USA), and samples were separated on a 4–12% gradient Bis-Tris gel (cat. number NP0335BBOX, ThermoFisher Scientific, Waltham, MA, USA) and transferred to a PVDF membrane (cat. number 162-0218, Bio-Rad Laboratories, Inc., Hercules, CA, USA). The membrane was blocked in blocking buffer containing 5% non-fat milk in TBS-T (20 mM Tis-HCl pH 7.4, 150 m NaCl, 0.1% Tween-20) for one hour at room temperature followed by incubation with rabbit anti-SLC25A46 (1:1000 dilution; cat. number 12277-1-AP, Proteintech, Rosemont, IL, USA) diluted in blocking buffer overnight at 4 °C with agitation. Following incubation with primary antibody, the membrane was washed three times in TBS-T and then incubated with IRDye 800CW Donkey anti-Rabbit IgG secondary antibody (926-32213, 1:500, LI-COR Biotechnology, Lincoln, NE, USA) and anti-tubulin hFA Rhodamine antibody (AbD22584, 1:500, Bio-Rad, Hercules, CA, USA) were used for immunofluorescence detection. Quantification of protein levels, normalized to tubulin, was performed using ImageJ software.

Analysis of mitochondrial morphology: Proband and two control fibroblasts were seeded with the same cell density in chamber slides and incubated overnight in complete media at 37 °C in cell culture incubator. The next day, the medium was removed, and cells were washed once in phosphate-buffered saline (PBS). Cells were treated with MitoTracker Orange CMTMRos (100 nM, M7510, ThermoFisher Scientific, Waltham, MA, USA); for 30 min at 37 °C, 5% CO_2_ incubator. The fibroblasts were washed twice in PBS. Cells were fixed with 4% paraformaldehyde for 15 min. After three washes in PBS, slides were coverslipped using Vectashield Mounting Medium with DAPI (H-1200, Vector Laboratories, Burlingame, CA, USA). Images (each group counts over 50 cells) were captured using a Nikon Eclipse 90i microscope in conjunction with NIS-Elements AR software (Nikon Instruments Inc., Melville, NY, USA). ImageJ macro (Mitochondrial Network Analysis) was used to analyze the mitochondrial morphology [9].

Statistical analysis: Statistical significance was assessed by a standard unpaired, two-tailed Student’s *t*-test; *p* value under 0.05 was considered significant. Results were analyzed with GraphPad Prism (v.8.3.0; GraphPad Software) and expressed as mean ± standard deviation (SD).

## 3. Results

### 3.1. Proband’s Phenotype

Following an uncomplicated pregnancy, the female proband was born at 39 weeks by spontaneous vaginal delivery to non-consanguineous parents, both in their late 20s. At birth, she weighed 6 pounds 9 ounces and had a routine 2-day hospital stay. During the first month of life, staring spells, motor nystagmus, and bilateral upper and lower limb flexion were noted. She was admitted to the hospital at 10 weeks of age due to lethargy and poor feeding, where she was ultimately intubated and treated for a UTI. During the 6-day hospital stay, additional testing was performed to investigate the neurological concerns. The 24-h EEG depicted bihemispheric multifocal epileptiform discharges, which did not correlate with the nystagmus observed. The proband was started on anti-epileptic medication. MRI and echocardiogram were normal.

The proband continued to have episodes of clenched fists and stiff upper limbs, which occurred about once or twice a day and lasted 5–10 s. She received physical and occupational therapy and was determined to have severe hypotonia and global developmental delay. At three months of age, the proband was hospitalized for inspiratory stridor, difficulty breathing, and poor weight gain. Following a supraglottoplasty and placement of NG tube, the proband was discharged, but was re-admitted days later for respiratory insufficiency, vomiting, and feeding intolerance. Nasopharyngeal reflux was seen by a swallow study, along with normal chest X-rays and GI series. A repeat swallow study was carried out a month later, around 4.5 months of age, and tracheal aspiration was noted, with impaired gag reflex. A G-tube replaced the NG tube. An EEG was recorded while the proband was awake and was within normal range with no seizures.

A repeat brain MRI was performed at five months of age that reported small, likely hypoplastic optic nerves. MRI spectroscopy revealed decreased N-acetyl aspartate (NAA) to choline ratio and decreased NAA to creatine ratio, with no lactate peak detected. Additional and extensive metabolic and genetic studies were normal: plasma amino acids, urine organic acid, plasma acylcarnitines, creatine kinase, chromosomal microarray, epilepsy panel, mitochondrial genome sequencing, and trio ES. A single detection of elevated lactate (3.5 mmol/L) and normal serum alanine put a mitochondrial disorder high on the differential.

At six months of age, she was admitted for presumed gastrointestinal illness and fevers and eventually intubated due to respiratory distress. At this age, she was less than the first percentile in weight, at the fourth percentile in height, and at the second percentile for head circumference. During this hospitalization, abnormal awake and asleep EEG were reported: occipital slowing and spikes indicating focal disturbance of cerebral functioning with epileptogenic potential as well as both hemispheres demonstrating diffuse cortical irritability with epileptogenic potential. A repeat EEG was performed a week later that supported the previous findings and together suggested a moderate encephalopathy with cerebral dysfunction in bilateral posterior regions and focal epileptogenic potential. Brain MRI and spectroscopy were then completed and showed abnormal signal development in the central/posterior brainstem with areas of symmetric T2 hyperintensity and diffused restriction in the globus pallidus and thalami. Cerebral spinal fluid was more plentiful around the brain leading to more prominent cerebral sulci and mildly larger ventricles. As observed previously, decreased NAA to creatine ratio was seen; however, a lactate peak was now visible. Collectively, these results were felt to be consistent with a mitochondrial disorder, particularly Leigh syndrome. The proband continued to experience respiratory distress and ultimately respiratory failure, secondary to the presumed mitochondrial disorder, that led to her early death at seven months of age.

### 3.2. SLC25A46 Variant Detection and Confirmation

**Figure 1:**
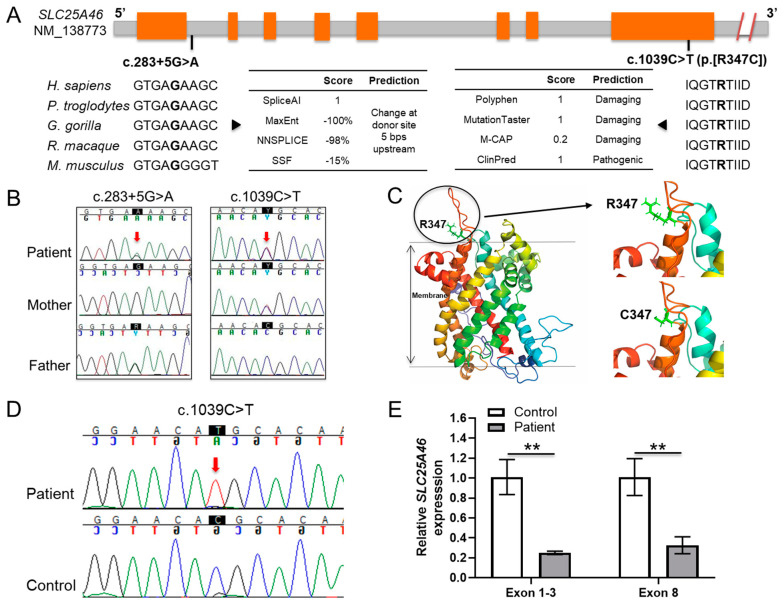
Identification and functional determination of the two novel *SLC25A46* variants in the proband. (**A**) Schematic of the *SLC25A46* variants. The nucleotide of the splice-region position is conserved and predicted to abolish the splice donor site (c.283+5G>A). The amino acid of the missense position is conserved and predicted to be damaging (c.1039C>T, p.[R347C]). Exons and introns are displayed by orange and gray blocks, respectively. (**B**) Sanger sequencing chromatogram for the family gDNA of the *SLC25A46* variants; (**C**) Structural model of SLC25A46 is shown as the six transmembrane helixes in rainbow colors (SLC25A46-whole). Residue R347 is located at the end of one helix outside the membrane shown in light green (R347) along with the variant C347. (**D**) Sanger sequencing chromatogram for the proband and control cDNA of the *SLC25A46* missense variant. (**E**) qRT-PCR analysis of the *SLC25A46* mRNA expression in the fibroblast cells of the proband and control using different primer pairs specific for exon 1-3 and exon 8 regions, ** *p* < 0.01.

Trio ES was performed clinically in 2017, which identified several variants of uncertain significance, none of which explained the phenotype. With no genetic diagnosis, the family reached out to The Manton Center for Orphan Disease Research and were enrolled in the Gene Discovery Core protocol (IRB number 10-02-0053). The clinical trio ES data was obtained and reanalyzed using the VExP pipeline [10]. Two novel trans *SLC25A46* variants (Figure 1A) were identified as strong candidates that matched the proband’s phenotype: maternally inherited c.1039C>T (p.Arg347Cys) and paternally inherited c.283+5G>A as shown in Figure 1B. Both variants are absent from the gnomAD database. The missense variant is predicted to be damaging by DANN, EIGEN, FATHMM-MKL, M-CAP, MutationAssessor, MutationTaster, PrimateAI, REVEL, and SIFT. The splice region variant was predicted to abolish the splice donor site by Alamut software: −99.0% (MaxEnt: −100.0%; NNSPLICE: −98.0%; SSF: −15.4%). Both mutations were confirmed by Sanger sequencing. Although not reported in the initial clinical ES, discovery of these variants by research reanalysis was communicated to the clinical laboratory. The clinical laboratory then performed ES reanalysis, which confirmed the presence of the trans SLC25A46 variants and resulted in a new report that classified both variants as likely pathogenic.

### 3.3. Protein Structure Analysis Affected by the Missense Variant

To assess the impact of the novel missense variant, a structural model of the SLC25A46 protein was built using the Robetta Tool (https://robetta.bakerlab.org/) accessed on 21 July 2021. In this model, SLC25A46 has six transmembrane helixes shown in rainbow colors (SLC25A46-whole) (Figure 1C). Residue R347 is located at the end of one helix outside the membrane (R347). The mutation R347C reduces the side chain volume and the positive charge (C347) of the protein. Such an alteration in charge might reduce the intermolecular or intramolecular interaction of SLC25A46. The presence of C347 might also lead to the formation of intermolecular disulfide bonds between SLC25A46. These structural studies suggest that the amino acid change and possible structural modification may interfere with the function of the SLC25A46 protein, rather than its expression or localization, as was seen with the donor splice region variant.

### 3.4. Transcription Analysis of the Donor Splice Region Variant

The variant in the donor splice region of intron 1 (c.285+5G>A) was predicted to alter the normal splicing of intron 1, thereby contributing to the pathogenicity of the disease. To investigate this hypothesis, cDNA was synthesized from total RNA extracted from proband and control fibroblasts, as described in the Methods section. *SLC25A46* cDNA sequences were amplified by PCR. When compared to control cDNA, Sanger sequencing identified a single missense allele in the patient (Figure 1D), indicating that the other had been degraded, possibly due to RNA decay. Additionally, levels of transcript (Figure 1E) were found to be significantly decreased (*p* < 0.01) in the proband by qRT-PCR analysis. These results demonstrate the pathogenicity of the splice region variant.

### 3.5. Altered Expression of SLC25A46 Mutant Protein

**Figure 2:**
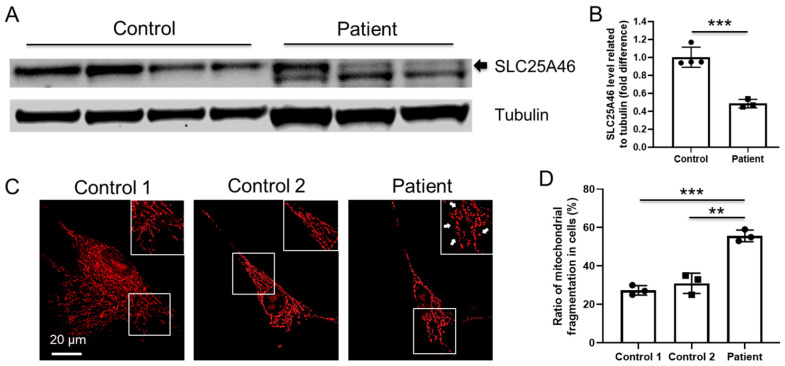
(**A**) Immunoblot analysis of total cell lysate from proband and two control fibroblasts using antibodies against SLC25A46 (46 kDa) and tubulin (55 kDa) as loading control. (**B**) Quantification of protein levels normalized to tubulin, *** *p* < 0.001. (**C**) Representative immunostaining images of Mitotracker (red) staining in control and proband fibroblasts. Insert plot is the magnified figure indicated by the white square. Arrow indicates the mitochondrial fragmentation in the proband cells. Scale bar=20 um. (**D**) Quantification of the mitochondrial fragmentation in fibroblast cells (** *p* < 0.001, *** *p* < 0.001, each group counts over 50 cells), compared to control.

To assess the impact of the missense (c.1039C>T, p.Arg347Cys) and donor splice region (c.283+5G>A) variants at protein level, proteins were extracted from proband and two control fibroblasts, and SLC25A46 expression was analyzed by Western blot (Figure 2A). The expected protein was seen as a 46 kDa band in both proband and age-matched control, with lower amounts (*p* < 0.001) present in the proband compared to control (Figure 2B).

### 3.6. Increased Mitochondrial Fragmentation in Proband’s Fibroblast Cells

SLC25A46 has been reported to localize in the mitochondrial outer membrane, where it plays important roles in mitochondrial architecture and dynamics [1,2,5]. To investigate the mitochondrial impact of these variants, we performed Mitotracker staining (Figure 2C and Figure S1) and found that proband’s fibroblast cells have more mitochondrial fragmentation compared with controls, suggesting alteration in the critical role of SLC25A46 in mitochondrial dynamics (Figure 2D).

## 4. Discussion

Strong evidence has emerged to show that mutations in mitochondrial proteins can be the underlying pathological cause of childhood- and adult-onset neurodegenerative disorders [11,12,13,14,15,16]. In addition to the two widely-studied mitochondrial proteins OPA1 and MFN2, mutations in *SLC25A46* have also been identified as a pathogenic cause in a spectrum of neurological and mitochondrial diseases [5,8,11,17,18]. Similar to previous *SLC25A46* case reports, the proband described here presented with a phenotype consistent with a mitochondrial disorder, including optic atrophy, brain MRI findings, elevated lactate levels, and early death (Table 1). The biallelic variants detected by our research reanalysis of clinical ES were not previously reported in population databases or the scientific literature. Our in vitro and in silico results suggest that these novel trans variants in *SLC25A46*, a missense (c.1039C>T, p.Arg347Cys) variant and a donor splice region (c.283+5G>A) variant, are the underlying pathological cause for the mitochondrial disorder in our deceased neonatal proband. The identification of these variants also highlights the importance of ES reanalysis to discover diagnoses in previously non-diagnostic genomic data. Indeed, hundreds of new genes and thousands of new variants are associated with different phenotypes every year [19,20,21]. The continued accumulation of genetic knowledge necessitates the regular reanalysis of genomic data.

In silico structural analysis of SLC25A46 suggested that the missense variant (c.1039C>T, p.Arg347Cys) could potentially affect the function of SLC25A46 by either altering or inhibiting its interaction with other proteins. This finding is supported by Abrams et al., who examined the effect of other missense variants located in the region within the 317–402 aa of SLC25A46 and proposed that pathogenic mutations in this gene either destabilize the protein or impact protein interactions [18]. Of the 10 missense variants reported thus far, seven occur in exon 8, which is also the location of this proband’s missense variant, further supporting this as a critical region for protein function.

Splice-site mutations can result in the complete skipping of one or more exons, retention of introns, or activation of a cryptic splice site within an exon or an intron [24,25,26]. A novel homozygous mutation (c.283+3G>T) in *SLC25A46* at the donor splice site very close to our proband’s mutation (c.283+5G>A) was reported in a patient with optic atrophy spectrum disorder [22]. The variant resulted in a shorter transcript generated from an alternative splice site within exon 1 and premature termination codon within exon 2 [22]. In our study, the splice region variant (c.283+5G>A) was strongly predicted to abolish the splice donor site by in silico analysis. Analysis of *SLC25A46* transcript levels by qRT-PCR confirmed the pathogenicity of the splice region variant.

The analysis of mitochondrial morphology in our proband cells revealed an increase in mitochondrial fragmentation, findings which have been previously described with *SLC25A46* mutations in Nguyen et al. [22]. However, loss of SLC25A46 has been linked to enlarged mitochondria with hyperfilamentous mitochondrial networks [1,27]. According to the study in Nguyen et al., the complete absence of SLC25A46 may cause the fragmentation of the mitochondrial network, whereas its reduction results in the mitochondrial hyperfusion [22]. In this study, the SLC25A46 reduction leads to increased mitochondrial fragmentation, which may be caused by a specific effect of the p.Arg347Cys missense mutation. The detrimental impact of these novel variants are further supported by the reduction in SLC25A46 as seen on Western blot. The proband’s phenotype also strongly aligns with other patients described, whose pathogenic mutations in *SLC25A6* alter mitochondrial dynamics.

## 5. Conclusions

In conclusion, we have identified two novel variants, one in the splice donor region of intron 1 (c.283+5G>A) and the other a missense variant (c.1039C>T, p.Arg347Cys) in the *SLC25A46* gene in a proband with Leigh syndrome-like presentation. Collectively, our findings suggest that these variants alter the amount and function of the protein, which are critical for maintaining mitochondrial dynamic, and are the cause of the neurological condition in this proband.

## Figures and Tables

**Table 1:** Genetic and clinical findings in individuals with *SLC25A46* splicing variants. NAA: N-acetyl aspartate.

	This Study	Nguyen et al. [22]	Braunisch et al. [23]
*SLC25A46* mutations	c.283+5G>A; c.1039C>T	c.283+3G>T (homozygous)	c.42C>G; c.462+1G>A
SLC25A46 proteins	Splicing defect; p.[Arg347Cys]	Splicing defect	p.[Tyr14Ter]; Splicing defect
Age of onset	<1 month	Birth	Birth
Age of death	7 months	7 days	1 day/18 days
Cause of death	Respiratory insufficiency	Respiratory insufficiency	Respiratory insufficiency
Optic atrophy	+	+	Unknown
Cerebellar or brainstem atrophy	+	+	+
Hypotonia	+	+	+
Other features	Global developmental delay, decreased NAA to choline ratio and decreased NAA to creatine ratio, with no lactate peak detected	Increase lactic acid level and lactate to pyruvate ratio, and decrease in cytochrome c oxidase activity	Pontocerebellar hypoplasia, respiratory defect, neurogenic lesion; loss of spinal motor neurons
Mitochondrial defects	Mitochondrial fragmentation	Mitochondrial fragmentation	Unknown

## Data Availability

The data presented in this study are available on request from the corresponding author.

## Supplementary Materials

The following are available online at https://www.mdpi.com/article/10.3390/jpm11121277/s1, Figure S1: Representative immunostaining images of Mitotracker (red) staining in control and proband fibroblasts (scale bar: 20 µm).
