# Supplementary figures and images for: Reanalysis of Exome Data Identifies Novel SLC25A46 Variants Associated with Leigh Syndrome
Source: J Pers Med. 2021 Dec 2;11(12):1277. doi: 10.3390/jpm11121277 (PMC8703603; doi:10.3390/jpm11121277)

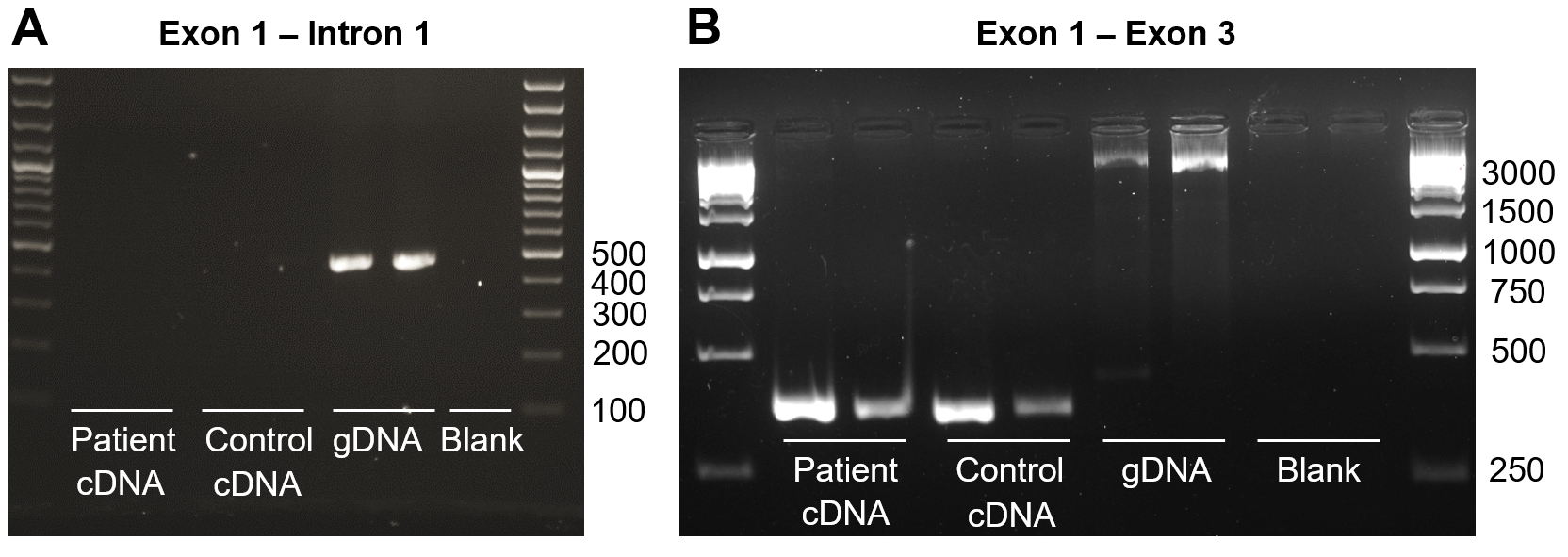

Supplement: Supplementary file 1 [file jpm-11-01277-s001.zip › Figure S1.tif]
